# Comparison of techno-functional and sensory properties of sponge cakes made with egg powder and different quality of powdered blood products for substituting egg allergen and developing functional food

**DOI:** 10.3389/fnut.2022.979594

**Published:** 2022-08-29

**Authors:** Tamás Csurka, Adrienn Varga-Tóth, Dorottya Kühn, Géza Hitka, Katalin Badak-Kerti, Boglárka Alpár, József Surányi, László Ferenc Friedrich, Klára Pásztor-Huszár

**Affiliations:** ^1^Department of Livestocks Products and Food Preservation Technology, Institute of Food Science and Technology, Hungarian University of Agriculture and Life Sciences, Budapest, Hungary; ^2^Doctoral School of Food Sciences, Hungarian University of Agriculture and Life Sciences, Budapest, Hungary; ^3^Department of Postharvest, Commerce, Supply Chain and Sensory Science, Institute of Food Science and Technology, Hungarian University of Agriculture and Life Sciences, Budapest, Hungary; ^4^Department of Grain and Industrial Plant Processing, Institute of Food Science and Technology, Hungarian University of Agriculture and Life Sciences, Budapest, Hungary

**Keywords:** animal blood, by-product, functional food, iron, iron deficiency anemia, sensory properties, sustainability, techno-functional properties

## Abstract

Animal blood is a valuable resource, which is usually not utilized in a value-added way by the industry like other animal by-products, even though it has plenty of benefits in terms of sustainability and human health, particularly against iron deficiency anemia. Animal blood is perfectly suitable for providing special functions, which are necessary for functional foods, and improving techno-functional properties based on the previous reports published in the literature. In this paper, egg powder was substituted by powdered animal blood products (whole blood powder, blood plasma powder, and hemoglobin powder) in sponge cake. Techno-functional and sensory properties (texture by texture profile analysis and three-point breaking test, water activity, dry matter content, and color) were instrumentally measured and then a sensory evaluation was carried out by unskilled panelists. Quality characteristics (texture, color, and dry matter content) were daily measured on the day of baking and then every 24 h for 3 additional days because freshly baked cakes are usually consumed within 3 days. Based on the results, powdered blood products are suitable for substituting the egg powder in sponge cakes and developing functional foods. Blood powders can increase the hardness, chewiness, and breaking force of cakes, giving them the ability to be stuffed with more fillings and molded into special shapes without compromising on the sensory characteristics. They can also increase the intensity of the cocoa flavor, which results in a richer, darker color without deceiving the consumers.

## Introduction

Animal blood is usually handled as hazardous waste and annihilated or in the best case used for producing feed raw material. However, it may play a role in sustainability in value-added further processing, and more sustainable products are preferred by consumers (1). Blood, which is obtained during the bleeding process when animals are slaughtered, accounts for a significant proportion of their live weight depending on the muscle mass: 7.6–8.3% in the case of cattle, 4.5–6% in the case of pig, 7.6–8.3% in the case of sheep, average 6.6% in the case of horse, and 5–10% in the case of poultry. Blood is a liquid connective tissue. Plasma is the intercellular part, in which the red blood cell (RBC) fraction or hemoglobin fraction is suspended. The RBC fraction attains its name from red blood cells because they make up the majority of this fraction, but it contains other blood cells like leucocytes, lymphocytes, and macrocytes, as well as many thrombocytes (2). Two-thirds of the blood take part in the circulatory system, and the residual one-third is present in the resting state and stored in organs like the spleen, liver, lungs, and other tissues (e.g., capillaries of skin). These blood storage organs are able to supply blood to the circulatory system in special cases (e.g., large blood loss). In many ways, animal blood can be an excellent raw material for the development and production of common and functional foods (3–7), and it is also suitable for the development of functional foods for allergen replacement purposes, as it is hypoallergenic (8, 9).

Functional foods are foods that contain higher than average amounts of ingredients, often artificially added, that have been shown to have a positive effect on health at the average normal consumption of the food (e.g., vitamin-enriched foods), or foods that contain lower than expected amounts of harmful ingredients at the average normal consumption of each food product (e.g., reduced-fat products). A functional food may also be a food intended for a specific group of consumers (e.g., people who suffer from a food allergy and bodybuilders). Functional foods are economically competitive and acceptable products that can be enriched with, for example, blood-derived bioactive peptides, protein (with good gelling and foaming properties), or hem-iron. Functional foods can play a major role in human nutrition during the current pandemic (10–13). A functional food can be a food that serves a specific need, such as the needs of consumers with egg allergies. IgE-mediated egg allergy affects up to 8.9% of children in developed countries (14).

It is hard to substitute egg white in bakery products because of its solubility, heat coagulation, foaming, and emulsifying properties (15). Animal blood plasma has already been used in bakery products and shown a similar effect to egg white in texture and appearance of cakes when blood plasma is used as an egg white alternative if blood plasma and egg white were in similar form (e.g. powder or liquid) and amount (16, 17). Plasma proteins contain mostly globular proteins (about 60 w/w% albumins and 40 w/w% globulins) and around 3–4 w/w% fibrinogen (18). According to recent studies, these are better for developing binding than meat powders, gelatin, wheat gluten, isolated soy proteins, and sodium alginate with calcium carbonate (19). In addition, other blood protein fractions (globulins, serum albumin, and hemoglobin) are better emulsifiers than an egg (20), in which egg white proteins contain 54–55 w/w% albumin, and egg yolk proteins contain 14 w/w% serum albumin, 41 w/w% glycoproteins, and 45 w/w% immunoglobulins (21). Investigating the effect of blood proteins on texture is important not only for substituting egg-allergenic ingredients or developing a harder, better texture, but also for improving the sustainability of animal slaughter and preventing or treating iron deficiency anemia.

The prevalence of anemia caused by iron deficiency is around 30% globally. Symptoms of anemia include physical and mental decline and miscarriage in pregnant women. Non-hem-iron absorption is <10%, but hem-iron absorption is 15–35%, and non-hem-iron is much more exposed to inhibitory effects due to the different metabolic pathways (22, 23).

A 100 g of porcine whole blood powder is able to cover the daily essential amino acid needs of an average 70 kg adult male with the exception of methionine (24), which can be supplemented with cereals. The iron content of porcine blood is 1,490.14 mg/kg, and in bovine blood, the iron content is 2,810.62 mg/kg expressed in terms of blood matter content (25, 26). In Russia and other post-Soviet states, blood chocolates and blood-colored candies are popular, especially for iron-deficient children. It is a good idea to give natural, well absorbable iron to children in the form of sweets or cakes. Due to our culture and trends, by-products such as blood are unlikely to become a major product in the foreseeable future, and the direct use of by-products on their own should not be forced. Therefore, the impact of blood and blood fractions on the techno-functional and organoleptic properties of foods should be investigated, and the results should be used to support and motivate industrial use.

Proteins have several different techno-functional properties. Hydration properties (water–protein interactions, such as hydration, water-holding capacity, dispersibility, solubility, and swelling) and surface properties (such as emulsification and foaming capacity) are very important basic properties. Rheological properties (protein–protein interactions, such as precipitation, gelation, and texturization) and sensory properties (such as taste (first of all, umami), texture, and color) are also basic properties but are affected by much more synergistic and divergent effects. Besides, techno-functional properties have a fifth category: the other properties (like adhesion, cohesion, film formation, etc.), which cannot be clearly classified under the previous ones (27). Blood plasma proteins are perfect cold binder agents (28), which can be helpful in the forming and handling of the batter before the heat treatment. In different food products, plasma proteins could improve the heat stability of the colloid system and decrease the heat treating loss (29–33). Blood products were successfully used in Kenya and Chile for improving the iron content of bakery products and in Russia in the case of sweets (34, 35).

The purpose of this paper was to briefly summarize the importance and role of blood in the development of functional (enriched with iron and/or protein and allergen-free) foods and investigate the effect of egg powder substitution in sponge cake with several types of blood powders (whole blood powder, hemoglobin powder, and plasma powder) on instrumentally measured sensory properties (color and texture) and techno-functional properties (pH, water activity, and dry matter content). Results were validated by a sensory evaluation of developed cakes. The objectives of this research were to compare the effect of egg powder, blood powder, blood plasma powder, and hemoglobin powder in cakes on quality-determining techno-functional and sensory properties (texture by texture profile analysis and three-point breaking test, water activity, dry matter content, color, appearance, taste and smell intensity, and liking) for substituting egg allergen by these valuable animal by-products.

## Materials and methods

### Materials

Whole blood powder and the powdered form of two main blood products (plasma powder and hemoglobin powder) were investigated for substituting egg powder and for developing functional product features (iron content). Thus, the protein content of the cake matrix was closely the same as the basic egg powdered in each sample type. Raw material specifications and a food nutrition database (26) were used for calculating the recipe. The amount of egg powder was calculated from average M-sized eggs, which is 58 g of weight. The cocoa-flavored sponge cake was prepared based on a common mix recipe, in which the protein content of the egg was substituted by different powdered blood products. The fat content of the egg was substituted by sunflower oil. Recipes are shown in Table 1. The water content of the egg was replaced by just water in case of each recipe. Determinative special ingredients, which were used during this investigation, were the follows:

- Hemoglobin powder 92B (Sonac Burgum B.V., Netherlands).
- Plasma powder 70B (Sonac Burgum B.V., Netherlands).
- Vepro 95 phf whole blood powder (Solvent Kereskedoház Zrt., Hungary).
- Egg powder (Capriovus Kft., Hungary).

**Table 1:** Ingredients [g or ml] in recipes of different investigated products.

**Ingredients**	**Cocoa flavored sponge with egg powder (control)**	**Cocoa flavored sponge with whole blood powder**	**Cocoa flavored sponge with hemoglobin powder**	**Cocoa flavored sponge with plasma powder**
Wheat plain flour (g)	300	300	300	300
Powdered sugar (g)	250	250	250	250
Margarin (g)	120	120	120	120
Milk with 2.8% fat (ml)	100	100	100	100
Cocoa powder with 15–16 w/w% fat content (g)	40	40	40	40
Baking powder (g)	12	12	12	12
Vanilla aroma (g)	2	2	2	2
Egg powder / Whole blood powder / Hemoglobin powder / Plasma powder (g)	28	14,16	13,8	18
Water (g)	88	92,06	92,66	88,8
Sunflower oil (g)	-	10,64	10,64	10,64

Replacement of fat in egg yolk is also necessary because fats and oils are important ingredients of bakery products. The major functions of lipids are to soften and tenderize the texture of the product by adding moisture, to make it richer and to improve the retention of quality. Sunflower oil was used in our research because it has no effect on the taste and smell of the product.

### Methods

#### Experimental design

In this paper, the results are presented according to a 4 × 4 full factorial experimental design for comparison of the effect of four different protein raw materials (egg powder, whole blood powder, blood plasma powder, and hemoglobin powder) with the same protein quantity and sample groups with four different storage times (0, 1, 2, and 3 days). The aim of the research plan was to detect the effect of different factors (sample type and storage time) on several techno-functional and sensory properties to investigate the quality of cakes.

#### Sample preparation

The batter was prepared in a bowl. Dry ingredients were mixed well, and then melted margarine was added into the dry mix, and all these were mixed thoroughly. Finally, the milk and water were added to the mix and then were further mixed using a household mixer. The raw foamy batter was filled into a metal tray and then heat-treated in a combined oven/steamer (Lainox VE051P Type LX, Italy) at 180°C with air mixing for 20 min. The sponge cake was sliced and stored for 3 additional days after the day of baking, because a fresh cake is expected to be stored for a maximum of 3–4 days before consumption.

The development of nutritional properties for the whole mass of all ingredients can be calculated according to the specifications and nutritional facts of ingredients. This theoretical development was only mathematically calculated in the case of 1% whole blood powder without any measurement, and no decomposition was assumed.

#### Storage experiment

It was also intended to carry out a storage experiment. From the day of production, the change in the quality of the sponge cake was examined during storage at room temperature for 3 days, since the product is expected to be consumed within this period of time at the latest in the case of fresh pastries and cakes. The storage conditions correspond to the average expected home storage conditions for the cakes, that is, at room temperature in imperfectly sealed (folded) plastic bags. In order to eliminate the effect of random changes in relative humidity in the air of the storage room, parallel samples were produced and sifted 1 day apart.

A 4-day storage period has been established because the texture and the dry matter content of the bakery products change in 4 days under room conditions according to the industrial experiments and previous studies (36, 37).

#### Texture measurement

The texture of sponge cakes was measured by using a Stable Micro System (SMS) TA. XT Plus texture analyzer (Stable Microsystem, United Kingdom). A computer controlled the movement of the upper rod. Different probes can be used to measure the different shear, compressive, and torsional stresses in different directions lasting for different time periods while investigating the behavior of the samples with this equipment.

Texture profile analysis (TPA) was carried out, and the following attributes were measured: (1) Hardness: the resistance [N] at maximum compression during the first compression. It is the force necessary to attain a given deformation. It represents the hardness of the sample at first bite. (2) Cohesiveness: The ratio [-] of positive force during the second compression cycle to that of the first one (downward strokes only). It indicates the strength of the internal bonds that make up the body of the sample. (3) Springiness: It is expressed as a ratio [mm mm-1] or percentage of a product's original height. Springiness is measured in several ways, but most typically, based on the distance of the detected height during the second compression divided by the original compression distance. (4) Chewiness: The force [N] required to chew a solid sample till a steady state of swallowing (hardness multiplied by cohesiveness multiplied by springiness) (38, 39). Sponge cake samples were sliced into sections with 40 × 40 mm square floor area and 20 mm height. The probe was a p/75v steel cylinder plate. The pre-test speed of the probe was 10 mm s^−1^, and the test speed and post-test speed were 1 mm s^−1^. Maximal compression was 50% of sample height (10 mm), and it lasted 1 s. Then the sample was allowed to relax. The sampling frequency was 50/s. Each sample was measured three times.

A fracture test was carried out, and the breaking force was measured, which is the peak force that can be measured when the cake dough sample is split into two portions. In the case of cakes with a hard crust, the peak force can be measured at the time of the breaking of crust instead of the full dough. HDP/3PB three-point bend rig probe was used. The probe started on the surface of the samples and then moved across the sample with a 1 mm s^−1^ test speed.

The limitation of this investigation is that lipids can affect the texture of gels and heat-treated gels (40). However, the aim of this research was the substitution of egg allergen in sponge cake. This limitation was a compromise for the substitution of egg powder without any additives.

#### Color measurement

Minolta CR-400 (Konica Minolta, INC., Japan) chroma meter was used for the measurement of reflection colors. The measurement was based on the fact that any color can be generated by the mixture of three colors defined by light wavelength. The ratio of these three different wavelength lights is plotted in a coordinate system called CIELAB color space. The color coordinates can be numbered, facilitating the analysis of colors. In the case of blood, there is a relationship between the red color (and chroma) and the iron content and thereby between the physical and chemical attributes.

The instrument was calibrated with a standard white etalon. Each sample was measured three times on a white plate with the same illumination. The measured attributes were as follows: redness/greenness (a^*^), yellowness/blueness (b^*^), and brightness (L^*^). Chroma (C^*^) was calculated according to the following equation:

Equation (1): $C^{*}=\sqrt{a^{*2}+b^{*2}}$ (41).

Hue angle was also an important color attribute, which was calculated according to the following equation:

Equation (2): $h_{ab}=arctan\frac{b^{*}}{a^{*}}$ (42).

#### Water activity measurement

Water activity was measured by using Novasina LabMaster-aw neo-type instrument (Novasina AG, Switzerland) that requires a very small sample amount and can fully control the temperature between 0 and 60°C during the measurement. Measurements were performed at room temperature to control the integrity of samples for relevant data detection. Each sample group was measured two times.

#### Dry matter content measurement

About 3–5 g of sample was measured by using a Kern ABJ-NM/ABS-N (Kern & Sohn GmbH, Germany) analytical balance and placed in an open Petri dish. Then the samples were dried at 120°C until a constant mass was obtained in a laboratory drying oven (Labor Muszeripari Muvek, Hungary). The samples were cooled in a desiccator, and then their residual mass was measured by using an analytical balance. Each sample was measured three times.

#### Sensory evaluation

A sensory evaluation was carried out in a lab equipped with adequate facilities (without confounding factors and with panel members separated in time and/or space). An online questionnaire was filled out by the panel members during the evaluation. The samples for the evaluation of this parameter were the analytical samples with an extra “placebo” sample, which was the same as the egg powdered sample but was colored with activated carbon. Vegetable activated carbon is an allowed food additive marked by E 153 according to one of the strictest regulations for food additives (the Regulation (EC) No 1333/2008 of the European Parliament and of the Council of 16 December 2008 on food additives) (43). Thanks to the active carbon, this egg powdered sample group had the same color as the whole blood powdered and hemoglobin powdered samples for the naked eye. Thus, it was possible to examine the perception of the product texture and taste without influencing the preconceptions of the color. The latter condition was necessary because a placebo/nocebo effect was found in earlier investigations. It means that taste perception depends on visual perception. Specifically, darker and/or more saturated color can mean a stronger taste and higher nutrition content for consumers (44–46). Panel members were informed that sponge cakes, which they taste, may contain blood, blood plasma, or extra cocoa (There was no sample with extra cocoa). They were informed that they may have got similar samples or different samples, but each panel member got the same five samples in a different sequence. Samples were labeled by a random three-digit number code, and each panel member got the samples in a different sequence to eliminate the effect of successive tasting, because the previous sample can influence the opinion of the next sample. Panel members were provided water to naturalize between the samples. The panel consisted of 33 panelists: 17 men and 16 women. The average age of panelists was 29.8 years, while the minimum age was 20 years and the maximum age was 56 years. The panel members were common consumers and not experts. Panel members had to test the samples and rank objectively the flexibility, crumbling, stickiness, dryness, intensity of cocoa smell, and intensity of cocoa flavor, as well as subjectively the smell, taste, and texture in category scales with five levels in a descriptive test. They could write a comment about the samples and rank the samples based on their preferences.

#### Statistical analysis

The measurement results were evaluated by applying IBM SPSS statistic v25 (IBM Corp., Armonk, NY) (47) and Microsoft Excel 365 version: 2010 (build: 13328.20356) software. A full factorial design was used, and results were statistically evaluated in three parts to detect the effect of raw material and storage time 1) on instrumentally measured texture parameters in the first analysis, 2) on color parameters in the second analysis, and 3) on sensory attributes in the third analysis. Dry matter content and water activity provided trend-like results. These two attributes could not provide a statistically significant difference. Multivariate analysis of variance (MANOVA) was carried out, to compare the means of different sample groups of the related variables (48). The normality of residuals was checked by Shapiro–Wilk test in the case of texture and color attributes and D'Agostino's K-squared test (49) in the case of sensory attributes. The normality of residuals for sensory attributes was not completely adequate for MANOVA, but in order to evaluate the results, MANOVA was carried out for all of the three attribute groups. According to Levene's test, the homogeneity of variances was adequate for the MANOVA in the case of color attributes and some texture attributes (hardness, chewiness, and breaking force). MANOVA is robust enough to test the equality of covariance matrices; hence, in the case of attributes with no adequate homogeneous covariance matrices according to the results of Levene's test, Box's M test (50) was carried out. In the case of each measured attribute analyzed in one MANOVA, the degree of freedom was the same, and differences in the maximal and minimal standard deviation values of the compared sample groups to the power of two were <2. So, homoscedasticity was adequate for MANOVA in the case of each texture, color, and sensory attribute. The value of the unexplained variance rate (Wilks's lambda) was evaluated. The homogeneous groups were separated by Tukey's HSD *post-hoc* test.

## Results and discussion

The effect of cereal proteins on the amino acid composition of blood and their worldwide consumption makes a sponge cake a perfect model product to study the effect of powdered blood products that are used as a substitute for egg powder in batter and cakes. In order to technologically design the structure of the cake, it is essential to understand the structure of the foams. As a result of mechanical stress (mixing), the foaming agents (protein emulsifiers) create a foam with an airy structure, the volume of which increases rapidly in the first stage of the whipping, and then gradually this growth slows down; finally, the air bubbles grow and merge when exceeding the critical value and the foam collapses (51, 52). Air absorption of batter depends on beater speed, geometric properties, surface tension, and viscosity of the batter (53). The amount and type of emulsifiers affect the bubble structure and distribution, which influence the quality of the final bakery product (54). Generally, emulsifiers may be proteins or lipids. Protein emulsifiers are responsible for mechanically strong viscoelastic films, and lipids enable weaker films to counteract changes in interfacial tension when the interface is perturbed (52). Protein-emulsifier interactions affect the rheology of emulsions according to recent studies (55–57). The dispersion medium of the foams can be liquid or solid, while the dispersed phase can only be gaseous. In the case of sponge cake batter, the proteins of the egg white (ovalbumin, conalbumin, ovomucoid, globulin, and ovomucin) are usually responsible for the formation of the foam structure. Under strong mechanical action, gas bubbles are dispersed in the liquid egg, the water-soluble proteins are denatured and then adsorbed on the surface of the colloidal solution, and the bubbles are surrounded by the formed film layer. Since albumin-type proteins typically make up the majority of proteins in the blood, especially in plasma, the blood is similarly capable of forming a colloidal structure. Foaming is affected by the following factors: pH and salinity. Flour and sugar are responsible for foam stability and foam hardness of bakery products. The discharged sponge flan is essentially a solid (58, 59). If the egg is substituted in a bakery product, the fat content of the egg also needs to be substituted.

### Texture

The normality of residuals was checked by Shapiro–Wilk test [hardness: W(160) = 0.993, *p* = 0.65; springiness: W(160) = 0.967, *p* = 0.05; cohesiveness: W(160) = 0.977, *p* = 0.08; chewiness: W(160) = 0.994, *p* = 0.734; breaking force: W(160) = 0.986, *p* = 0.102], and the homogeneity of variances was checked by Levene's test attributes [hardness: *F*_(15,144)_ = 2.137, *p* = 0.011; chewiness: *F*_(15,144)_= 1.272, *p* = 0.227; breaking force: *F*_(15,144)_= 1.904; *p* = 0.027] and Box's M test (springiness, cohesivity: *n* = 10, *N* = 160) to confirm that MANOVA could be carried out.

Sponge cakes were made with egg powder, whole blood powder, hemoglobin powder, and blood plasma powder as a protein source and stored for 3 days. The texture of the cakes in all the sample groups was similar to a common sponge cake. The nominal differences between the results of different sample groups were not significant, but a significant difference was found in the case of each measured texture attribute. The effect of the raw material and storage time on texture attributes was investigated by the first MANOVA. The overall MANOVA result was significant for the sample type, that is, the protein source in the used raw material, (Wilks's lambda: 0.009, *p* < 0.001) and the storage time (Wilks's lambda: 0.028, *p* < 0.001). The two-way interaction between the sample type and storage time was also significant (Wilks's lambda: 0.054, *p* < 0.001). The results of Wilks's lambda tests indicate a strong relationship between the factors and dependent variants, but the effect for the sample type was the strongest.

Different sample types could be separated from each other. Tukey's *post-hoc* test could significantly (*p* = 0.05) separate three groups based on hardness: the cakes with egg powder were the softest with an average hardness value of 6.92 N, cakes with whole blood powder and blood plasma powder were similar with average hardness values of 9.1 and 9.4 N, respectively, and cakes with hemoglobin powder were the hardest with the average hardness value of 11.9 N. It can be explained by the fact that albumin content is similar in egg and blood plasma and can develop a lighter batter with more air bubbles due to their foaming properties. Whole blood powder contains plasma proteins, but hemoglobin powder does not. If the aim of product development is a harder cake, which can show, for instance, a greater weight of filling or higher iron content, hemoglobin powder enrichment is an ideal choice. But, if the aim of product development is a nearly common sponge cake texture without egg allergen, blood plasma powder or whole blood plasma powder is the best choice. The hardness values of cakes made with 20– 40 g kg^−1^ egg in other studies were below these values, and were closer to the values of the cake made with egg powder (60–62). Based on springiness, Tukey's *post-hoc* test could significantly (*p* = 0.05) separate two different groups with nominally not much significant difference. The samples prepared with whole blood powder and egg powder were similar with average springiness values of 0.69 and 0.71, respectively, and these were different from samples prepared with hemoglobin powder and blood plasma powder which showed average springiness values of 0.75 and 0.77, respectively. Springiness values of cakes made with the egg from the previous studies were found to be 0.82, 0.56, and 0.85. The second result was closer to the value of the cake made with plasma powder and egg powder. The higher springiness values can be explained by the fact that using fresh eggs makes a better foam than using a powdered egg (60–62). In the case of chewiness, there was a significant difference (*p* = 0.05) between all sample groups, but the difference was not statistically significant. The samples with egg powder were less chewable with an average chewiness value of 4.01 N. Then the samples with whole blood powder showed an average chewiness value of 4.43 N. Cakes with blood plasma powder had a greater chewiness value (average 5.24 N), and cakes with hemoglobin powder was the most chewable (average 6.18 N). There was one comparable chewiness result, that is, 5.17 N (Rodriguez-García). It was closer to the cakes made with blood plasma powder. There was a significant difference (*p* = 0.05) between all sample groups with regard to the breaking force. This value best reflected the foaming ability of different protein sources because air bubbles in the cake dough weaken the structure of cakes and make it easier to break it. The results of chewiness and breaking force may play a role in product development like hardness values. Samples with egg powder were the easiest breakable with an average of 5.42 breaking force. Breaking force values (average 6.08 N) of samples with blood plasma powder were close to the values of egg powdered samples. The breaking force of samples with whole blood powder (average 7.31 N) and hemoglobin powder (average 8.17 N) was also significantly different (*p* = 0.05).

**Figure 1:**
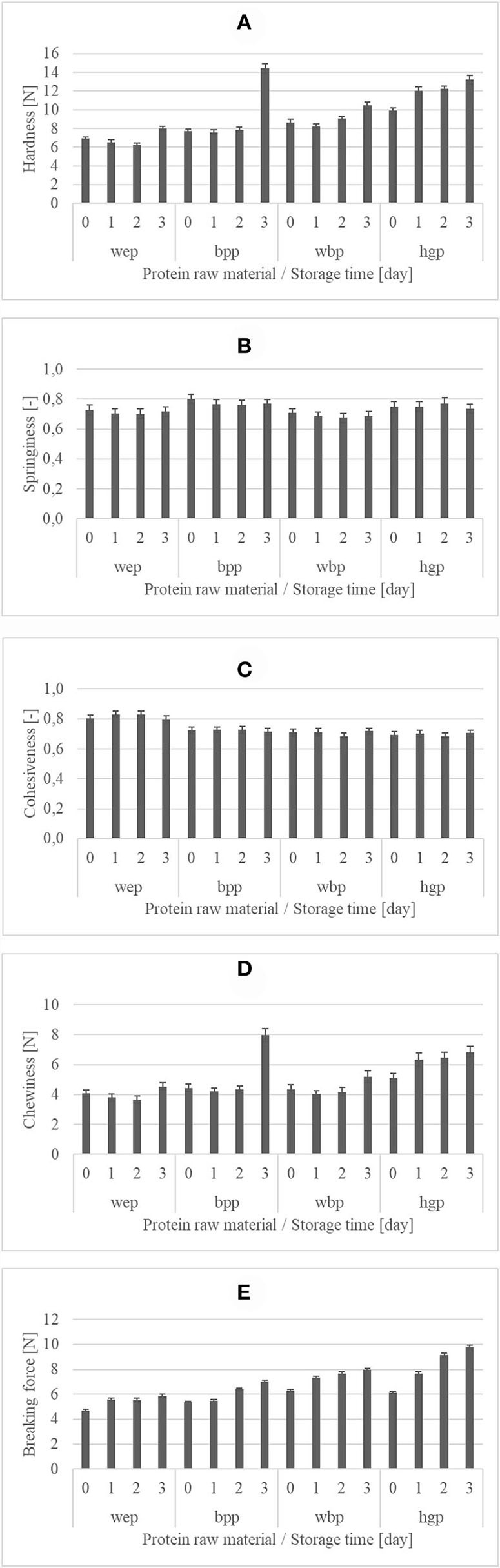
Texture attributes [**(A)** hardness [N]; **(B)** springiness [-]; **(C)** cohesiveness [-]; **(D)** chewiness [N]; **(E)** breaking force [N]] of sponge cake sample groups with different protein raw materials (wep, whole egg powder; bpp, blood plasma powder; wbp, whole blood powder; hgb, hemoglobin powder) and stored for different time periods (0 day – day of baking after cooling, 1 day, 2 days, and 3 days).

Storage time also had a significant effect on texture. However, this change was a nominally small difference between the texture attributes of different sampling times. Consumers often eat the cakes only 2 or 3 days after purchase. Based on these results, each cake composition can guarantee a constant quality until the third day after the baking day. In the case of hardness, a nominally significant change could be observed only between the samples prepared from blood plasma powder on the second and third storage days. Thus, the average hardness value of sample groups on the third day was 32% greater than the average hardness value of sample groups on the first and second days. Chewiness results were similar: There was a significant difference (*p* = 0.05) between the chewiness of sample groups on the third day and sample groups on other days, and there was no significant difference between the chewiness values of the sample groups from the starting day, and the first and second storage days. Average chewiness values increased by 34% for the third storage day from the average value of sample groups with 0, 1, and 2 days of storage time. Increasing hardness and chewiness and nearly stagnant springiness values during storage were reported in other studies as well (63–66). It can be explained by syneresis caused by drying, which had been significant on the second and third days. In the case of springiness and cohesiveness, there was no significant difference between the sample groups with different storage time periods. The effect of storage time is the clearest in the case of breaking force, which is related to the drying of the crust. Increasing crust hardness caused an increasing breaking force. There was a significant difference (*p* = 005) between all the sample groups with different storage time periods in case of breaking force, which is similar to the common cakes. The values of color attributes of sponge cake sample groups made with different protein raw material and stored for different times can be seen in Figure 1.

### Color

The normality of residuals was checked by Shapiro–Wilk test [a^*^: W(60) = 0.98, *p* = 0.442; b^*^: W(60) = 0.984, *p* = 0.644; L^*^: W(60) = 0.989, *p* = 0.878; C^*^: W(60) = 0.968, *p* = 0.121; Hue angle: W(60) = 0.978, *p* = 0.367], and the homogeneity of variances was checked by Levene's test attributes [a^*^: *F*_(19, 40)_= 2.172, *p* = 0.222; b^*^: *F*_(19, 40)_= 1.809, *p* = 0.057; L^*^: *F*_(19, 40)_= 1.370, *p* = 0.197; C^*^: *F*_(19, 40)_= 2.982, *p* = 0.002; Hue angle: *F*_(19, 40)_= 1.916, *p* = 0.042] to confirm that MANOVA could be carried out.

**Figure 2:**
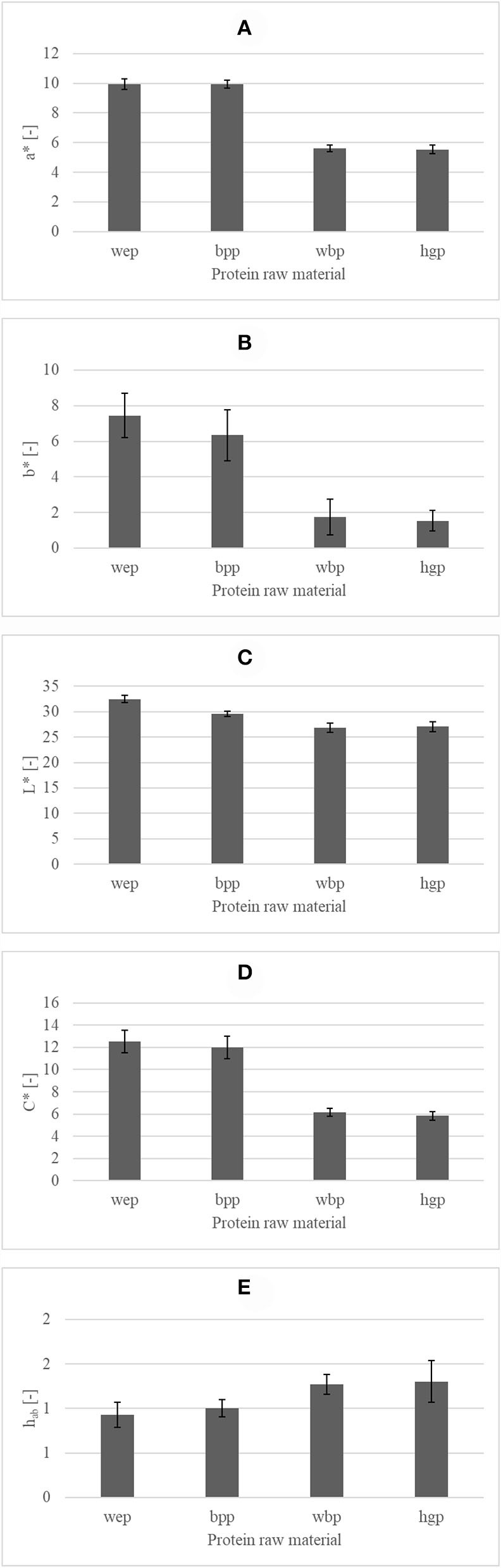
Color attributes [**(A)** a* - redness-greenness [-]; **(B)** b* - yellowness-blueness [-]; **(C)** L* - lightness [-]; **(D)** C* - chroma [-]; **(E)** h_ab_ - hue angle] of sponge cake sample groups with different protein raw materials (wep, whole egg powder; bpp, blood plasma powder; wbp, whole blood powder; hgb, hemoglobin powder).

As it was expected, the colorization of heat-treated hemoglobin had the main effect on the color attributes, which could be observed in all the color attributes (L^*^, a^*^, b^*^, and C^*^). The color difference between the different sample types could be seen by the naked eye, and a significant difference was found based on the calculated total color differences. The values of color attributes of sponge cake sample groups made with different protein raw materials can be seen in Figure 2. The effect of the raw material and storage time on color attributes was investigated by the first MANOVA. The overall MANOVA result was significant for the sample type, that is, used protein source raw material (Wilks's lambda: 0.004, *p* < 0.001), and the storage time (Wilks's lambda: 0.012, *p* < 0.001). The protein raw material better determined the dependent variables. The effect of storage time also was significant, but the nominal differences between the average color values of different sample groups were very small (1-2 values on the color coordinate scales), while some color values were doubled between two different sample types. Furthermore, a particular pattern could not be observed in the color result based on the storage time, since color attributes fluctuated in different ways. The two-way interaction between the sample type and storage time was also significant (Wilks's lambda: 0.019, *p* < 0.001). The effect of interaction was weaker, but relatively stronger. According to the results of Tukey's *post-hoc* test, two different groups could be significantly (*p* = 0.05) separated based on redness-greenness coordinates; egg powdered and plasma powdered samples were redder with an average 9.94 a^*^ value, while the whole blood powdered and hemoglobin powdered samples, which contained hem-iron, were less red with 5.74 a^*^ value. Three groups could be significantly (*p* = 0.05) separated based on lightness; the lightest samples were the egg powdered samples with 32.5 L^*^ value, the next ones were the plasma powdered samples with 29.59 L^*^ value, and the darkest were the whole blood powdered and hemoglobin powdered samples with an average of 26.97 L^*^ value. These results clearly indicate that the addition of hem-iron can cause black colorization in the heat-treated products rather than red colorization. In the case of other types of the food matrix, non-linear darkening was observed as a result of hemoglobin addition (67).

Because of this, hem-iron can be utilized in originally dark-colored products or beside adequate marketing tools for marketing the high nutritional value of the product. Based on yellowness-blueness coordinates, three groups could be significantly (*p* = 0.05) separated. The hemoglobin powdered and whole blood powdered samples were less yellow with a 1.63 b^*^ value, the plasma powdered samples were more yellow with a 6.34 b^*^ value, and the egg powdered samples were the most yellow with a 7.44 b^*^ value. Two groups could be significantly (*p* = 0.05) separated based on chroma: one group contained the samples with hem-iron with an average 6.11 C^*^ value and the other group contained the plasma powdered and egg powdered samples with a 12.24 C^*^ value. Two groups could be significantly (*p* = 0.05) separated based on Hue angle: one group contained the samples with hem-iron with an average 1.33 Hue angle value, and the other group contained the plasma powdered and egg powdered samples with a 0.99 Hue angle value. Hue angle and chroma have two factors: redness-greenness and yellowness-blueness coordinates. The significant differences observed in the Hue angle reflect redness-greenness. It could be said that sample groups, which contained hem-iron, were globally similar, and egg powdered and blood plasma powdered sample groups were also slightly similar in the case of color attributes.

### Water activity and dry matter content

The results of water activity measurement (not shown) were difficult to evaluate because of the large standard deviation. Water activity results of crumb (inner side of cake) from samples on the day of baking and on the first day of storage were between 0.52 and 0.66 in the case of all products and depended on the distance of the analytical sample part from the crust.

The dry matter content of sample groups with different raw materials was not significantly different. The average dry matter content of each sample group was nearly 25 w/w%, except for the sponge cakes made with blood plasma powder which showed 23 w/w% dry matter content. Although the difference was not significant, it can be explained by the outstandingly good foaming and water binding capacity of blood plasma, and this foam structure may lose less water during the baking process. Most of the sample groups typically changed similarly: cakes became wetter from the humidity of the air between the day of baking and the first storing day and then started to dry until the second day because of the syneresis, due to the aging of colloid systems. It was interesting that dry matter content continuously increased in the case of the samples with whole blood powder from the first storing day, but it decreased again in the case of other sample groups from the second storing day, as these got more wetter. This observation may be connected with the syneresis of different types of colloid systems.

### Sensory evaluation

The normality of residuals was checked by D'Agostino's K-squared test, and the homogeneity of variances was checked by Box's M test (objective flexibility, objective crumbing, objective stickiness, objective dryness, objective cocoa smell intensity, objective cocoa flavor intensity, preference smell, preference color, preference taste, preference texture, and preference overall impression: *n* = 33, *N* = 165) to confirm that MANOVA could be carried out.

The results of sensory evaluation had a relatively great standard deviation, so these were hard to statistically evaluate in case of objective as well as subjective properties. The descriptive statistics of sensory properties is listed in Table 2. Significant differences and lack of significant differences between sample groups are marked in Table 2. Based on the results of MANOVA, the effect of the sample type on the dependent variables was not significant (Wilks's lambda: 0.587, *p* < 0.001). It means that a greater part of the dependent variables could not be explained by the differences in the sample types than part could be. The panel could not identify the differences between the different sample groups, except for a few properties according to Tukey's *post-hoc* test. The few exceptions, in which different groups could be significantly (*p* = 0.05) separated, were the following: (1) based on objective flexibility, two groups were identified (group 1: hemoglobin powdered, whole blood powdered, and egg powdered samples; group 2: whole blood powdered, egg powdered, blood plasma powdered, and black colored egg powdered samples); (2) based on objective cocoa flavor, two groups were identified (group 1: blood plasma powdered, whole blood powdered, egg powdered, and hemoglobin powdered samples; group 2: whole blood powdered, egg powdered, hemoglobin powdered, and black colored egg powdered samples); (3) based on color preference, three groups were identified (group 1: egg powdered and blood plasma powdered samples; group 2: blood plasma powdered, hemoglobin powdered, and black colored egg powdered samples; group 3: hemoglobin powdered, black colored egg powdered, and whole blood powdered samples); and (4) based on texture preference, two groups were identified (group 1: hemoglobin powdered, whole blood powdered, egg powdered, and blood plasma powdered samples; group 2: whole blood powdered, egg powdered, blood plasma powdered, and black colored egg powdered samples). This result can be explained by the fact that panelists probably defined the perceived softness (inversely proportional to hardness) as the flexibility of the sponge cake samples. So, differences in the hardness of different sponge cake types, which were instrumentally measured by TPA, could be slightly detected by the unskilled panelists. It was interesting that sample groups made with egg powder were slightly different. This difference is due to the preconception caused by the color difference. Interestingly, the results of objectively evaluated flexibility were not fully expressed in the preference for texture. In contrast to TPA, this can hardly be explained by the similar foaming and texturizing properties of plasma and egg only. The small difference in objective cocoa flavor and color preference can be explained by the instrumentally measured color results, because the color of foods can influence consumers. This assumption could be confirmed by the fact that control egg powdered sponge cakes and “placebo” black colored egg powdered samples were assessed differently in the case of each sensory property. This difference was self-evidently completely significant (*p* = 0.05) in case of color preference, for a part of the panel members significant (*p* = 0.05) in case of objective flexibility, objective cocoa flavor and texture preference, and trend-like in case of other sensory attributes. According to the comments of panelists, sometimes extra cocoa flavor or blood taste and/or smell could be noticed in the “placebo” samples. The majority of panelists could notice the blood or iron flavor in sponge cakes made with whole blood powder. It was interesting that sponge cakes made with blood plasma powder were recognized as control samples or more sweet egg powdered samples by a few panelists. It could be explained by the light color and higher salt content of these cakes, because salt has a synergist effect on sweetness.

**Table 2:** Descriptive statistics (mean [-], standard deviation [-], and sample number [-]) and homogeneous subsets of results of sensory properties (objective flexibility, objective crumbing, objective stickiness, objective dryness, objective cocoa smell intensity, objective cocoa flavor intensity, preference smell, preference color, preference taste, preference texture, and preference overall impression) of different sample groups (samples with blood plasma powder, samples with egg powder, black colored samples with egg powder, samples with hemoglobin powder, and samples with whole blood powder).

**Sample type**		**Mean**	**Std. deviation**	**N**	**Homogeneous subsets**
Flexibility - objective	Samples with blood plasma powder	3.67	1.19	33	b
	Samples with egg powder	3.24	1.06	33	a,b
	Black colored samples with egg powder	3.73	0.91	33	b
	Samples with hemoglobin powder	2.88	1.24	33	a
	Samples with whole blood powder	3.09	0.84	33	a,b
	Total	3.32	1.10	165	
Crumbing - objective	Samples with blood plasma powder	2.52	1.00	33	a
	Samples with egg powder	3.03	1.05	33	a
	Black colored samples with egg powder	2.61	1.06	33	a
	Samples with hemoglobin powder	3.18	1.10	33	a
	Samples with whole blood powder	3.24	1.23	33	a
	Total	2.92	1.12	165	
Stickiness - objective	Samples with blood plasma powder	2.58	0.94	33	a
	Samples with egg powder	2.67	0.99	33	a
	Black colored samples with egg powder	2.64	0.96	33	a
	Samples with hemoglobin powder	2.52	0.94	33	a
	Samples with whole blood powder	2.85	0.91	33	a
	Total	2.65	0.94	165	
Dryness - objective	Samples with blood plasma powder	2.21	0.99	33	a
	Samples with egg powder	2.52	1.03	33	a
	Black colored samples with egg powder	1.97	1.05	33	a
	Samples with hemoglobin powder	2.12	0.99	33	a
	Samples with whole blood powder	2.03	0.99	33	a
	Total	2.17	1.01	165	
Cocoa smell - objective	Samples with blood plasma powder	3.00	0.87	33	a
	Samples with egg powder	2.97	0.98	33	a
	Black colored samples with egg powder	3.30	1.26	33	a
	Samples with hemoglobin powder	3.27	1.01	33	a
	Samples with whole blood powder	2.97	1.21	33	a
	Total	3.10	1.07	165	
Cocoa flavor - objective	Samples with blood plasma powder	2.94	1.17	33	a
	Samples with egg powder	3.09	0.98	33	a,b
	Black colored samples with egg powder	3.73	0.94	33	b
	Samples with hemoglobin powder	3.36	1.19	33	a,b
	Samples with whole blood powder	3.03	1.24	33	a,b
	Total	3.23	1.13	165	
Smell - preference	Samples with blood plasma powder	3.09	1.13	33	a
	Samples with egg powder	3.24	0.87	33	a
	Black colored samples with egg powder	3.58	1.12	33	a
	Samples with hemoglobin powder	3.33	1.05	33	a
	Samples with whole blood powder	3.12	1.08	33	a
	Total	3.27	1.06	165	
Color - preference	Samples with blood plasma powder	3.42	1.17	33	a,b
	Samples with egg powder	3.06	1.06	33	a
	Black colored samples with egg powder	4.09	0.84	33	b,c
	Samples with hemoglobin powder	4.03	0.95	33	b,c
	Samples with whole blood powder	4.12	1.02	33	c
	Total	3.75	1.09	165	
Taste - preference	Samples with blood plasma powder	3.12	1.11	33	a
	Samples with egg powder	3.33	1.05	33	a
	Black colored samples with egg powder	3.55	1.09	33	a
	Samples with hemoglobin powder	3.36	1.06	33	a
	Samples with whole blood powder	2.82	1.18	33	a
	Total	3.24	1.11	165	
Texture - preference	Samples with blood plasma powder	3.24	1.17	33	a,b
	Samples with egg powder	3.18	0.85	33	a,b
	Black colored samples with egg powder	3.73	1.07	33	b
	Samples with hemoglobin powder	2.97	1.19	33	a
	Samples with whole blood powder	3.03	0.92	33	a,b
	Total	3.23	1.07	165	
Overall impression - preference	Samples with blood plasma powder	3.42	1.20	33	a
	Samples with egg powder	3.33	0.92	33	a
	Black colored samples with egg powder	3.82	1.01	33	a
	Samples with hemoglobin powder	3.45	1.06	33	a
	Samples with whole blood powder	3.24	1.06	33	a
	Total	3.45	1.06	165	

This assumption could be confirmed by the fact that control egg powdered sponge cakes and “placebo” black colored egg powdered samples were assessed differently in the case of each sensory property. This difference was self-evidently completely significant (*p* = 0.05) in case of color preference, for a part of the panel members significant (*p* = 0.05) in case of objective flexibility, objective cocoa flavor and texture preference, and trend-like in case of other sensory attributes.

### Development of nutritional properties

In addition to the effect of egg allergen substitution by powdered blood products on techno-functional and sensory properties, the development of nutritional properties has to be mentioned. The added iron content in the case of porcine/bovine whole blood powder was 1.5 mg / 2.9 mg. Iron added with 1 w/w% whole porcine/bovine blood powder covers 18.8% / 36.3% of the iron requirement of an average man, or 8.3% / 16.1% of the iron requirement of an average woman. So, an increase in iron content was also significant in the case of whole blood powder and hemoglobin powder enrichment. Hemoglobin powder contains almost just hemoglobin from blood proteins. Hem-origin iron content of sponge cake made with whole blood powder and hemoglobin powder can help to prevent and may play a role in the treatment of childhood iron deficiency anemia in an acceptable form for children.

Pure plasma powder does not contain hem-iron, but it contains the albumin fraction of blood proteins, which is responsible for the texture developing effect of whole blood powder. To further investigate this effect, a separate examination with plasma powder is preferred.

## Conclusion

Based on the results of this study, powdered blood products are suitable substituents for the egg in cakes. Although the difference between sponge cakes made with egg powder and blood products is measurable and perceptible. Some quality attributes of the product rather improved as well as the new products were completely egg-free. A good example of the improved properties is that the texture was harder and more chewable in case of cakes made with blood products instead of egg as well as the color was darker and more saturated in case of cakes made with whole blood and hemoglobin powder. The darker and more saturated colors made the samples appear to contain more cocoa. Sponge cakes made with blood plasma powder are more similar to common sponge cakes made with egg powder, thanks to the similar albumin content and being free from hem-iron, but this benefit also means a great disadvantage in the view of nutritional value. Dry matter content and water activity were at a desirable level in all the sample groups. Due to drying and changes in colloidal structure, all the sample groups became harder and more chewable during storage. However, this change was so small that it can only be determined with instrumental measurement. It did not affect the final quality of the product. The differences between texture and sensory properties of sponge cakes made with egg powder and different blood origin powders could not or could just barely be noticed by unskilled panelists (consumers). An interesting finding was that the unskilled panelists have a different perception of the whole sample because of the different preconceptions based on the different appearances in the case of two sample groups with the same ingredients. Concerning all attributes, there was no best cake, but only cakes which fit better for purpose. If the purpose is substituting egg powder with the smallest color and texture change, the best choice is the blood plasma powder based on the results of this study. But besides coloring matters, egg powder can be substituted by whole blood powder and hemoglobin powder because consumers cannot feel the difference between the different cakes. If the purpose is to develop a harder, less breakable cake, which can take more fillings or can stand rougher handling, the best choice would be the cake made with hemoglobin powder. Whole blood and hemoglobin enrichments may be acceptable in cakes and sweetness by children, and a harder, less breakable sponge cake can stand special formatting in case of production of desserts for children.

## Data availability statement

The raw data supporting the conclusions of this article will be made available by the authors, without undue reservation.

## Ethics statement

Ethical review and approval was not required for the study on human participants in accordance with the local legislation and institutional requirements. Written informed consent from the participants or participants legal guardian/next of kin was not required to participate in this study in accordance with the national legislation and the institutional requirements.

## Author contributions

TC and AV-T contributed to conception. TC conceived and designed the analysis, contributed to statistical analysis, and wrote the first draft of the manuscript. TC and DK collected the data and performed the analysis. AV-T and KP-H contributed to manuscript revision, read, and approved the submitted version. GH supervised the storage technology aspect of the work. KB-K supervised the bakery science aspect of the work. BA supervised the nutritional science aspect of the work. JS and LF supervised the livestock science and technology aspect of the work. All authors contributed to the article and approved the submitted version.

## Funding

This research was supported by the Hungarian University of Agriculture and Life Sciences, Doctoral School of Food Sciences (Budapest, Hungary). This research was funded by the Ministry of Innovation and Technology within the framework of the Thematic Excellence Program 2021, National Defense, National Security Subprogram (TKP2021-NVA-22).

## Conflict of interest

The authors declare that the research was conducted in the absence of any commercial or financial relationships that could be construed as a potential conflict of interest.

## Publisher's note

All claims expressed in this article are solely those of the authors and do not necessarily represent those of their affiliated organizations, or those of the publisher, the editors and the reviewers. Any product that may be evaluated in this article, or claim that may be made by its manufacturer, is not guaranteed or endorsed by the publisher.

## References

[1] Floros JD Newsome R Fisher W Barbosa-Cánovas GV Chen H Dunne CP. Feeding the world today and tomorrow: the importance of food science and technology. Compr Rev Food Sci Food Saf. (2010) 9:572–99. 10.1111/j.1541-4337.2010.00127.x 33467827

[2] Liu XQ Yonekura M Tsutsumi M Sano Y. Physicochemical properties of aggregates of globin hydrolysates. J Agric Food Chem. (1996) 44:2957–61. 10.1021/jf9505786

[3] Duarte RT Carvalho Simões MC Sgarbieri VC. Bovine blood components: fractionation, composition, and nutritive value. J Agric Food Chem. (1999) 47:231–6. 10.1021/jf9806255 10563877

[4] Hsieh YHP Ofori JA. Blood-derived products for human consumption. Revel Sci. (2011) 1:14.21.

[5] Ofori JA Hsieh YHP. The Use of Blood and Derived Products as Food Additives. In Food additive. New York, NY; San Francisco, CA; London: Intech Open Access Publisher (2012). p. 299–56.

[6] Toldrá F Aristoy MC Mora L Reig M. Innovations in value-addition of edible meat by-products. Meat Sci. (2012) 92:290–6. 10.1016/j.meatsci.2012.04.004 22560456

[7] Bah CS Bekhit AEDA Carne A McConnell MA. Slaughterhouse blood: an emerging source of bioactive compounds. Compr Rev Food Sci Food Saf. (2013) 12:314–31. 10.1111/1541-4337.12013

[8] Csurka T Szücs F Csehi B Friedrich LF Pásztor-Huszár K. Substitution of milk allergen ingredient by blood plasma powder in custard with different sweeteners. Prog Agric Eng Sci. (2021) 17:77–85. 10.1556/446.2021.30010

[9] Csurka T Szücs F Csehi B Friedrich LF Pásztor-Huszár K. Analysis of several techno-functional and sensory attributes upon egg allergen ingredient substitution by blood plasma powder in sponge cake. Prog Agric Eng Sci. (2021) 17:87–98. 10.1556/446.2021.30011

[10] Galanakis CM. Separation of functional macromolecules and micromolecules: from ultrafiltration to the border of nanofiltration. Trends Food Sci Technol. (2015) 42:44–63. 10.1016/j.tifs.2014.11.005

[11] Galanakis CM. The food systems in the era of the coronavirus (COVID-19) pandemic crisis. Foods. (2020) 9:523. 10.3390/foods9040523 32331259 PMC7230343

[12] Galanakis CM Aldawoud T Rizou M Rowan NJ Ibrahim SA. Food ingredients and active compounds against the Coronavirus disease (COVID-19) pandemic: a comprehensive review. Foods. (2020) 9:1701. 10.3390/foods9111701 33233560 PMC7699782

[13] Galanakis CM. Functionality of food components and emerging technologies. Foods. (2021) 10:128. 10.3390/foods10010128 33435589 PMC7826514

[14] Osborne NJ Koplin JJ Martin PE Gurrin LC Lowe AJ Matheson MC. HealthNuts Investigators. Prevalence of challenge-proven IgE-mediated food allergy using population-based sampling and predetermined challenge criteria in infants. J Allergy Clin Immunol. (2011) 127:668–76. 10.1016/j.jaci.2011.01.039 21377036

[15] Shepherd IS. Yoel RW. “Cake emulsions”. Ch. 5. In: Friberg S editor. Food Emulsions. New York, NY, USA: Marcel Dekker. (1976). p. 215–75.

[16] Johnson LA Havel EF Hoseney RC. Bovine plasma as a replacement for egg in cakes. Cereal Chem. (1979) 56:339–42.

[17] Lee CC Johnson LA Love JA Johnson S. As an egg white substitute in cakes'. Cereal Chem. (1991) 68:100–4.

[18] Putnam F. The Plasma Proteins V3: Structure, Function, and Genetic Control, 2nd ed. Volume III. Bloomington, IN: Elsevier (2012).

[19] Lu GH Chen TC. Application of egg white and plasma powders as muscle food binding agents. J Food Eng. (1999) 42:147–51. 10.1016/S0260-8774(99)00112-0

[20] Caldironi HA Ockerman HW. Incorporation of blood proteins into sausage. J Food Sci. (1982) 47:405–8. 10.1111/j.1365-2621.1982.tb10091.x24746974

[21] Jolivet P Boulard C Chardot T Anton M. New insights into the structure of apolipoprotein B from low-density lipoproteins and identification of a novel YGP-like protein in hen egg yolk. J Agric Food Chem. (2008) 56:5871–9. 10.1021/jf800321m 18558702

[22] Liberal Â Pinela J Vívar-Quintana AM Ferreira IC Barros L. Fighting iron-deficiency anemia: innovations in food fortificants and biofortification strategies. Foods. (2020) 9:1871. 10.3390/foods9121871 33333874 PMC7765292

[23] Egyed A. Carrier mediated iron transport through erythroid cell membrane. Br J Haematol. (1988) 68:483–6. 10.1111/j.1365-2141.1988.tb04241.x 3377990

[24] Ockerman HW Hansen CL. Animal Byproduct Processing and Utilization. Boca Raton, London, New York, Washington, D.C: CRC Press. (2000) 10.1201/9781482293920

[25] Sorapukdee S Narunatsopanon S. Comparative study on compositions and functional properties of porcine, chicken and duck blood. Korean J Food Sci Anim Resour. (2017) 37:228. 10.5851/kosfa.2017.37.2.228 28515647 PMC5434210

[26] USDA United United States Department of Agriculture Agricultural Research, Service, Nutrient Data, Laboratory,. (2018). USDA National Nutrient Database for Standard Reference, Release 29. Available online at: https://fdc.nal.usda.gov/ (accessed November 17, 2018).

[27] Yada RY Jackman R Smith JL. Protein Structure-Function Relationships in Foods. Glasgow: Blackie Academic and Professional (1994). 10.1007/978-1-5904615-2670-4

[28] Herrero AM Cambero MI Ordonez JA De la Hoz L Carmona P. Plasma powder as cold-set binding agent for meat system: Rheological and Raman spectroscopy study. Food Chem. (2009) 593: 493–9. 10.1016/j.foodchem.2008.07.084

[29] Cofrades S Guerra MA Carballo J Martin F Colmenero FJ. Plasma protein and soy fiber content effect on bologna sausage properties as influenced by fat level. J Food Sci. (2000) 65:281–27. 10.1111/j.1365-2621.2000.tb15994.x

[30] Jarmoluk A Pietrasik Z. Response surface methodology study on the effects of blood plasma, microbial transglutaminase and κ-carrageenan on pork batter gel properties. J Food Engg. (2003) 60:327–34. 10.1016/S0260-8774(03)00055-4

[31] Pietrasik Z Jarmoluk A Shand P.J. Effect of non-meat proteins on hydration and textural properties of pork meat gels enhanced with microbial transglutaminase. LWT Food Sci Technol. (2007) 40:915–20. 10.1016/j.lwt.2006.03.003

[32] Seideman SC Smith GC Carpenter ZL Dill CW. Plasma protein isolate and textured soy protein in ground beef formulations. J Food Sci. (1979) 44:1032–5. 10.1111/j.1365-2621.1979.tb03439.x

[33] Suter DA Sustek E Dill CW Marshall WH Carpenter ZL. A method for measurement of the effect of blood protein concentrates on the binding forces in cooked ground beef patties. J Food Sci. (1976) 41:1428–32. 10.1111/j.1365-2621.1976.tb01188.x

[34] Andago A Lamuka JIAMP Nduati R Kangemi K. Development of A Bovine Blood Enriched Porridge Flour for Alleviation of Anaemia Among Young Children in Kenya. (2015).

[35] Walter T Hertrampf E Pizarro F Olivares M Llaguno S Letelier A. Effect of bovine-hemoglobin-fortified cookies on iron status of schoolchildren: a nationwide program in Chile. Am J Clin Nutr. (1933) 57:190–4. 10.1093/ajcn/57.2.190 8424387

[36] Soukoulis C Yonekura L Gan HH Behboudi-Jobbehdar S Parmenter C Fisk I. Probiotic edible films as a new strategy for developing functional bakery products: The case of pan bread. Food Hydrocol. (2014) 39:231–42. 10.1111/j.1750-3841.2006.00005.x 25089068 PMC4007592

[37] Pohjanheimo TA Hakala MA Tahvonen RL Salminen SJ Kallio HP. Flaxseed in breadmaking: Effects on sensory quality, aging, and composition of bakery products. J Food Sci. (2006) 71:343–8. 10.1016/j.foodhyd.2014.01.023 25089068 PMC4007592

[38] De Huidobro FR Miguel E Blázquez B Onega E A. comparison between two methods (Warner–Bratzler and texture profile analysis) for testing either raw meat or cooked meat. Meat Sci. (2005) 69:527–36. 10.1016/j.meatsci.2004.09.008 22062992

[39] Nishinari K Fang Y Rosenthal A. Human oral processing and texture profile analysis parameters: Bridging the gap between the sensory evaluation and the instrumental measurements. J Texture Stud. (2019) 50:369–80. 10.1111/jtxs.12404 31008516

[40] Paraskevopoulou A Kiosseoglou V. Texture profile analysis of heat-formed gels and cakes prepared with low cholesterol egg yolk concentrates. J Food Sci. (1997) 62:208–11. 10.1111/j.1365-2621.1997.tb04401.x

[41] Tomasevic I Tomovic V Ikonic P Rodriguez JML Barba FJ Djekic I. Evaluation of poultry meat colour using computer vision system and colourimeter: is there a difference? Br Food J. (2019) 121:1078–87. 10.1108/BFJ-06-2018-0376

[42] Anese M Sovrano S Bortolomeazzi R. Effect of radiofrequency heating on acrylamide formation in bakery products. European Food Research and Technology. (2008) 226:1197–203 10.1007/s00217-007-0693-x

[43] European Union. Regulation (EC) No 1333/2008 of the European Parliament and of the Council of 16 December 2008 on Food Additives. 1078–87.

[44] Forgács A Bóna E Csíkos T. Az ízpreferenciák, ízaverziók és ételfóbiák pszichológiai vonatkozásai. Magyar Belorvosi Archívum. (2017) 70:300–10. Available online at: http://real-j.mtak.hu/11358/6/MBA%202017%206.pdf

[45] Földváry M. Színkommunikáció: Élelmiszer és színe.(2015) 1.4.3. Available online at: http://www.szinkommunikacio.hu/14_07.htm (accessed September 21, 2021).

[46] Spence C. On the psychological impact of food colour. Flavour. (2015) 4:21. 10.1186/s13411-015-0031-3

[47] *IBM SPSS Statistics for Windows Version 25.0*. Armonk, NY: IBM Corp.

[48] Huberty CJ Olejnik S. Applied MANOVA and discriminant analysis (Vol. 498). Hoboken, NJ: John Wiley & Sons. (2006).

[49] D'Agostino RB. Transformation to normality of the null distribution of g1. Biometrika. (1970) 57:679–81. 10.1093/biomet/57.3.679

[50] Box GE. A general distribution theory for a class of likelihood criteria. Biometrika. (1949) 36:317–46. 10.1093/biomet/36.3-4.317 15402070

[51] Carlin GT. A microscopic study of the behavior of fats in cake batters. Cereal Chem. (1944) 21:189–99.

[52] Coke M Wilde PJ Russell EJ Clark DC. The influence of surface composition and molecular diffusion on the stability of foams formed from protein/surfactant mixtures. J Colloid Interface Sci. (1990) 138:489–504. 10.1016/0021-9797(90)90231-C

[53] Handelman AR Conn JF Lyons JW. Bubble mechanics in thick foams and their effects on cake quality. Cereal Chem. (1961) 38:294–305.

[54] Brooker BE. The stabilisation of air in cake batters-the role of fat. Food Struct. (1993) 12:285–96.

[55] Dickinson E Hong ST. Influence of water-soluble nonionic emulsifier on the rheology of heat-set protein-stabilized emulsion gels. J Agric Food Chem. (1995) 43:2560–6. 10.1021/jf00058a002

[56] Dickinson E Yamamoto Y. Viscoelastic properties of heat-set whey protein-stabilized emulsion gels with added lecithin. J Food Sci. (1996) 61:811–6. 10.1111/j.1365-2621.1996.tb12208.x

[57] Chen J Dickinson E. Viscoelastic properties of protein-stabilized emulsions: Effect of protein– surfactant interactions. J Agric Food Chem. (1998) 46:91–7. 10.1021/jf970536c 10554201

[58] Awuchi CG Igwe VS Echeta CK. The functional properties of foods and flours. Int J Adv Acad Res. (2019) 5:139–60.

[59] Turbin-Orger A Della Valle G Doublier JL Fameau AL Marze S Saulnier L. Foaming and rheological properties of the liquid phase extracted from wheat flour dough. Food Hydrocoll. (2015) 43:114–24. 10.1016/j.foodhyd.2014.05.003

[60] Rodríguez-García J Puig A Salvador A Hernando I. Optimization of a sponge cake formulation with inulin as fat replacer: structure, physicochemical, and sensory properties. J Food Sci. (2012) 77:C189–97. 10.1111/j.1750-3841.2011.02546.x 22250810

[61] Guadarrama-Lezama AY Carrillo-Navas H Pérez-Alonso C Vernon-Carter EJ Alvarez-Ramirez J. Thermal and rheological properties of sponge cake batters and texture and microstructural characteristics of sponge cake made with native corn starch in partial or total replacement of wheat flour. LWT. (2016) 70:46–54. 10.1016/j.lwt.2016.02.031

[62] Hosseini H Bolourian S Shahidi F. Extending the shelf-life of sponge cake by an optimized level of jujube fruit flour determined using custom mixture design. Br Food J. (2019) 121:12. 10.1108/BFJ-07-2019-0489

[63] Salehi F Kashaninejad M. Texture profile analysis and stress relaxation characteristics of quince sponge cake. J Food Meas Charact. (2018) 12:1203–10. 10.1007/s11694-018-9734-3

[64] Sahi SS Alava JM. Functionality of emulsifiers in sponge cake production. J Sci Food Agric. (2003) 83:1419–29. 10.1002/jsfa.1557

[65] Gupta M Bawa AS Semwal AD. Effect of barley flour incorporation on the instrumental texture of sponge cake. Int J Food Prop. (2009) 12:243–51. 10.1080/10942910802312082

[66] Salehi F Kashaninejad M. Modeling of moisture loss kinetics and color changes in the surface of lemon slice during the combined infrared-vacuum drying. Inf Proc Agric. (2018) 5:516–23. 10.1016/j.inpa.2018.05.006

[67] Oellingrath IM Sslinde E. Color, pigment and iron content of meat loaves with blood, blood emulsion, or mechanically deboned meat added. J Food Sci. (1985) 50:1551–5 10.1111/j.1365-2621.1985.tb10531.x

